# Gut bacterial community profile of the endemic catfish *Arius manillensis* from Pasig River, Philippines

**DOI:** 10.1128/mra.00370-26

**Published:** 2026-07-10

**Authors:** Clara Patricia T. Lirio, Elourjane Eunice D. Albino, Kizzy Kaye S. Nisnisan, Arizaldo E. Castro

**Affiliations:** 1 Biological Sciences Department, College of Science, De La Salle University–Dasmariñas, Dasmariñas City, Cavite, Philippines; 2 College of Professional Graduate Studies, De La Salle University–Dasmariñas, Dasmariñas City, Cavite, Philippines; Montana State University, Bozeman, Montana, USA

**Keywords:** gut microbiome, freshwater microbiology, *Arius manillensis*, bacterial communities, fish, metagenomics

## Abstract

The Pasig River is a highly urbanized waterway, yet the microbial ecology of its native fauna remains poorly understood. This study provides the first report of the gut bacterial community of the catfish *Arius manillensis*, revealing bacterial taxa and underscoring the need to study host-associated microbiomes in urban aquatic ecosystems.

## ANNOUNCEMENT

*Arius manillensis* is an endemic catfish (1) inhabiting the Pasig River, a highly urbanized waterway with documented water quality shifts (2, 3), and ongoing anthropogenic influences (4, 5). Despite these conditions, microbial studies on native fauna remain limited, underscoring the need to examine host-associated microbiomes in urbanized tropical rivers.

**Fig 1:**
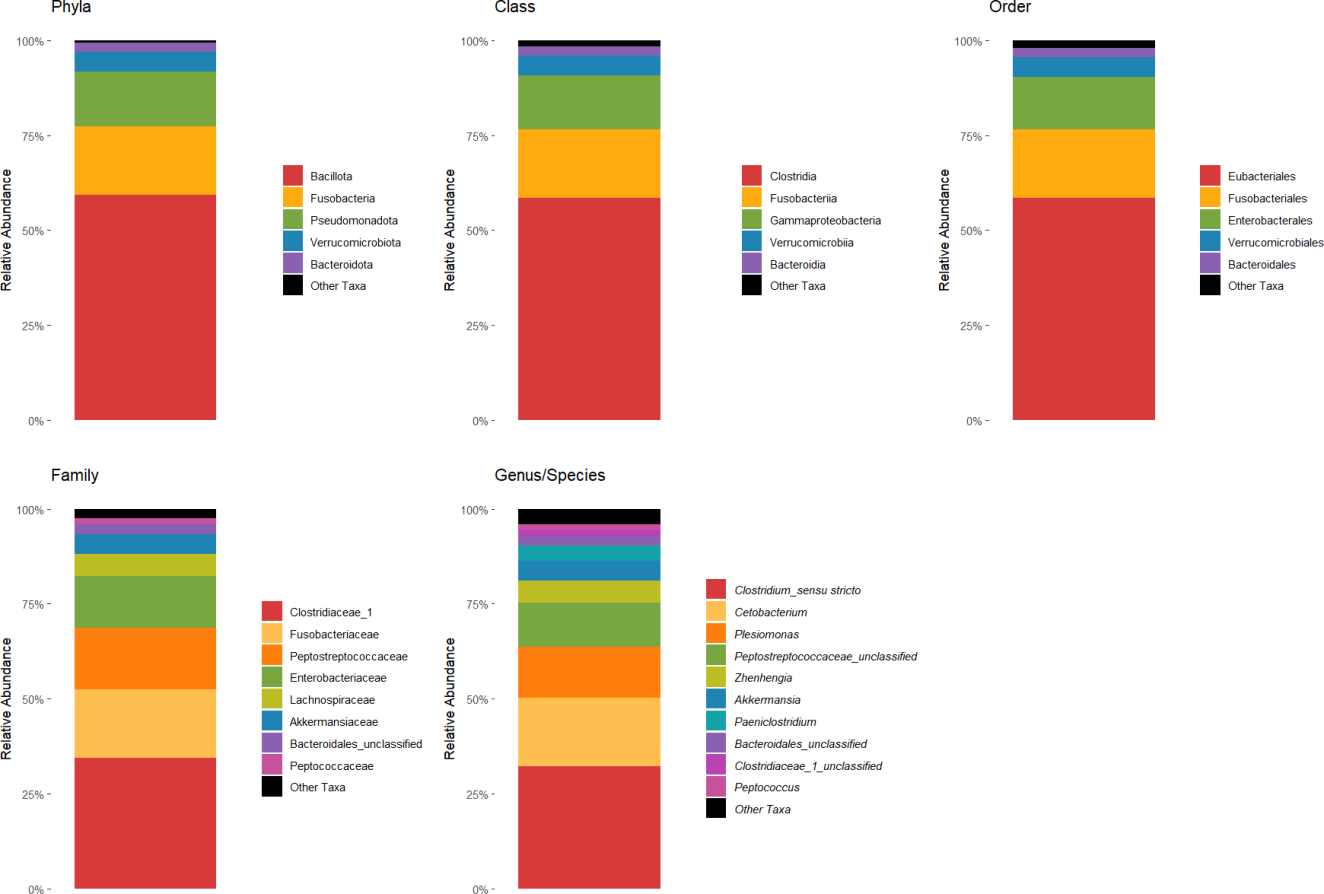
Gut bacterial community profile of *A. manillensis.*

Three *A. manillensis* individuals were collected on 29 October 2025 from Pasig River (14°32'21.0"N 121°05'51.0"E). Gut DNA was extracted using the PrimeWay Genomic II DNA Extraction Kit (1st BASE, Singapore). All three gut tissue samples were processed for metagenomic *16S rRNA* gene sequencing (V3–V4 region) using 341F (5′-CCTACGGGNGGCWGCAG-3′) and 805R (5′-GACTACHVGGGTATCTAATCC-3′) primers, yielding three sets of paired-end files. Following library preparation with MOPC-purified primers, sequencing was performed on a MiSeq i100 Plus platform (300 bp paired-end). Raw reads underwent quality filtering (Q ≥ 20) via fastp (0.24.1) (6), while quality control assessments were performed using FastQC (0.12.0) (7). Triplicate paired-end files were pooled for operational taxonomic unit (OTU) clustering (97% similarity) via mothur (1.48.0) (8) and taxonomic assignment using the SILVA 138.2 database (9). Lacking clean-water controls, environmental correlations were based on literature comparison. Relative abundances across five taxonomic levels (phylum, class, order, family, and genus/species) were calculated and visualized as stacked bar plots (Fig. 1).

The gut microbiome is dominated by Bacillota (59.2%), Fusobacteria (18.1%), Pseudomonadota (14.5%), Verrucomicrobiota (5.2%), and Bacteroidota (2.5%). Given the use of the V3–V4 region, genus-level assignments are putative due to limited resolution for closely related taxa. Sequences are predominantly mapped to *Clostridium sensu stricto* (32.1%), *Cetobacterium* (17.7%), *Plesiomonas* (13.9%), Peptostreptococcaceae_unclassified (11.5%), *Akkermansia* (5.1%), and *Zhenhengia* (5.8%). *Clostridium*, the most abundant genus-level classification, is associated with carbohydrate fermentation and nutrient processing. Sequences putatively assigned to *Cetobacterium* are linked to vitamin B12 synthesis (10) and gut stability. Reads mapped to *Plesiomonas*, an urban freshwater taxon (11), may reflect environmental stress, while sequences mapped to *Akkermansia* suggest potential mucin-degrading activity (12). These predicted anaerobic groups likely contribute to fermentative metabolism, short-chain fatty acid production, and carbohydrate degradation essential for intestinal energy homeostasis.

Predicted functional profiles using METAGENassist (13) suggest enrichment of bacteria putatively involved in dehalogenation (40.2%), ammonia oxidation (35.4%), and chitin degradation. Identified dehalogenating groups (*Pseudomonas*, *Acinetobacter*, *Bacillus*, *Rhodococcus*) suggest microbial community adaptation to halogenated organic compounds in the urbanized river. Bacteria associated with ammonia oxidation indicate involvement in nitrogen metabolism, consistent with elevated river ammonia levels. Predicted chitin-degrading groups (including members of Clostridia, Gammaproteobacteria, and Verrucomicrobiota) suggest the presence of chitin-rich organic compounds.

The dominance of anaerobic fermenters, nutrient-cycling groups, and taxa characteristic of urban waterways suggests that the *A. manillensis* gut microbiome is shaped by both host-associated processes and the environment of the Pasig River (14). This study provides the first publicly accessible gut microbiome profile for *A. manillensis*. The presence of bacterial groups linked to urban environments and predicted detoxification functions highlights the species’ potential as a biological indicator for modified tropical river systems.

## Data Availability

The sequence data generated for this project have been deposited in the National Center for Biotechnology Information Sequence Read Archive (NCBI SRA) under BioProject PRJNA1398810 with accession numbers SRR36689010, SRR36689009, and SRR36689008.
